# Attenuation of Morphine Physical Dependence and Blood Levels of Cortisol by Central and Systemic Administration of Ramelteon in Rat

**Published:** 2015-05

**Authors:** Majid Motaghinejad, Ozra Motaghinejad, Pantea Hosseini

**Affiliations:** 1 Department of Pharmacology, School of Medicine, Iran University of Medical Sciences, Tehran, Iran;; 2 Department of Surgery, Faculty of Veterinary Medicine, University of Tehran, Tehran, Iran

**Keywords:** Ramelteon, Morphine, Dependency, Withdrawal syndrome, Cortisol

## Abstract

**Background:**

Chronic administration of morphine cause physical dependence but the exact mechanism of this phenomenon remains unclear. The aim of this study is the assessment of systemic and intracerebroventricular (*icv*) administration of ramelteon (a melatonin receptor agonist) on morphine physical dependence.

**Methods:**

88 adult male rats were divided into 2 major groups, namely “systematic” and “central” administration of ramelteon. In the first category, systemic administration of ramelteon at various dosages (10, 20, and 40 mg/kg) was assessed on dependent animals and withdrawal signs were compared with positive (received morphine and saline as systemic administration), negative control (saline) and group under treatment by ramelteon (40 mg/kg) groups. In the second category, central administration of ramelteon at various dosages (25, 50, or 100 μg,) was assessed on dependent animals and withdrawal signs were compared with the positive control (received morphine and saline as *icv*) and negative control (saline) groups, and the group under treatment by ramelteon (50 μg/5 μl/rat). On the test day, all animals received naloxone (3 mg/kg) and were observed for withdrawal signs. Total withdrawal score (TWS) was also determined. Finally, to evaluate the stress level of dependent rats, blood cortisols were measured.

**Results:**

Central administration of ramelteon in all doses and systemic administration in high doses attenuate withdrawal syndrome in comparison with the dependent positive control group (P<0.05). Both central and systemic administrations of ramelteon can attenuate the blood cortisol level in comparison with the dependent positive control group (P<0.05).

**Conclusion:**

In conclusion, we found that central administration of ramelteon attenuated morphine withdrawal symptoms and cortisol level as a stress marker.

## Introduction

Long-term administration of morphine has been accepted as the standard method for pain relief in patients with acute pain.^1^ Long-term use of morphine, which is characterized by physical dependency, result in the onset of withdrawal syndrome when opioid usage is suddenly discontinued.^2^ However; the particular mechanism which can vindicate dependence and withdrawal symptoms of morphine are not clear. Previous studies suggested that neurochemical dysfunction in nucleus accumbens followed by systemic morphine administration was the main reason for dependency to morphine. These studies suggested that, dopaminergic and serotonergic systems in nucleus accumbens have a critical role in dependence to morphine.^3^^,^^4^

Melatonin, a hormone that control circadian rhythms (e.g. sleep and wake cycle), is made by a small gland in the brain known as the pineal gland.^5^ Recent studies demonstrate the critical role of melatonin in neuromodulation in nucleus accumbens. This hormone plays a significant role in the regulation of various neural processes such as pain perception.^6-8^ Previous studies have characterized the interaction between pineal melatonin and the serotonergic function. These studies have indicated that melatonin has potential effects on the modulation of serotonergic and dopaminergic cell bodies, which may relate to the mode of action of melatonin and its behavioral effects.^9-11^ Moreover, based on the results of additional studies, melatonin modulates the release of Ach in the nucleus accumbens and can alter functions of reward system in the rats. Melatonin receptors have an antinociceptive activity in melatonin in formalin-induced pain response. This study showed that the antinociceptive action of melatonin is mediated by the release of opioid-like peptides in particular of the brain, such as nucleus accumbens.^8^ Pretreatment with melatonin significantly attenuated the anxiolytic-like effects of rats withdrawn from repeated cocaine administration, which could be used as a novel treatment for cocaine dependency.^12^^,^^13^ In cocaine dependent mice, physical withdrawal syndromes are inhibited by melatonin.^14^ On the other hand, morphine withdrawal is characterized by an increase in the activity of hypothalamus-pituitary-adrenocortical (HPA) axis and an increase in corticosterone secretion and blood cortisol levels, as corticosterone is responsible for stress and anxiety of the withdrawal period.^15^^,^^16^ Since mRNA expression is required for the synthesis of the CRH (corticotrophin releasing hormone) and opioid dependency increase mRNA expression, opioid dependency has a positive effect on the secretion of cortisol.^16^^,^^17^ Also, previous studies have indicated that melatonin has potential anxiolytic effects.^18^ Melatonin is effective in reducing the postoperative anxiety of morphine consumption.^19^ Recent data demonstrated anxiolytic properties of melatonin type-2 receptors.^18^ These results show that, melatonin has high potential for usage as an anxiolytic agent and could be used as stress modulating medication.

The aim of this study was to evaluate the possible role of intraperitoneal (*ip*) or intracerebroventricular (*icv*) pre-treatment with ramelteon, a melatonin receptor agonist, on morphine-induced withdrawal symptoms. In addition to the observable signs of morphine abstinence, for the assessment of ramelteon on anxiety and stress of withdrawal syndrome, blood cortisols were also measured.

## Materials and Methods

*The Animals*

88 adult male rats (200-250 grams) were obtained from Pasteur Institute of Iran and transferred to the animal lab of the central laboratory of Tehran University of Medical Sciences. The animals had free access to water and food and were kept in a controlled environment with a 12-hour light cycle and controlled room temperature of 22±3ºC. The rats were kept in the animal house for 7 days without any experimental challenge, in order to adapt them to their new conditions. All experimental procedures followed the guidelines on ethical standards for experiment on pain in animals and carried out according to a protocol approved by the local Animal Ethics Committee

*The Drug*

Morphine, naloxone and ramelteon and other chemical were purchased from Sigma (Aldrich Inc., St Louis MO, USA).

*The Experimental Groups*

Rats were randomly divided into 11 groups, namely:

- Group I, as “negative control group” (independent) received normal saline (1 ml/kg bw, *ip*) for 9 days.
- Group II, as****“positive control group for systemic administration of ramelteon” (dependent) received morphine with an increasing dosage (15-55 mg/kg *s.c*) for the first 5 days and were then received saline solution (1 ml/kg, *ip*) concurrently with the highest dose of morphine once a day from day 5 to 9.
- Groups III, IV and V received morphine with an increasing dosage (15-55 mg/kg* s.c* ) for the first 5 days and then ramelteon was injected with doses of 10, 20 and 40 mg/kg bw,* ip,* respectively and concurrently with morphine once in a day from days 5 to 9.
- Group VI, as****“positive control group for *icv* administration of ramelteon” (dependent) received morphine with an increasing dosage (15-55 mg/kg *s.c*) for the first 5 days and then were injected saline solution (1 ml/kg, *ip*) concurrently with the highest dose of morphine once in a day from day 5 to day 9.
- Groups VII, VIII and IX received morphine with an increasing dosage (15-55 mg/kg* s.c*) for the first 5 days and then ramelteon was concurrently injected with morphine at dosages of 25, 50 or 100 μg/5 μl/rat, *icv,* from day 5 to day 9, respectively.
- Groups X and XI were treated just by ramelteon (40 mg/kg bw,* ip*) or ramelteon (100 μg/5 μl/rat,* icv*) for 9 days.^20^^,^^21^

For the *icv *administration, a Hamilton syringe was used and connected to a length of PE-10 polyethylene tubing. Seeing the air bubble through the tube confirmed the injection efficacy. The volume of each infusion was 5 μl/site at a rate of 5 μl/ min for each rat.^22^

*Intracerebroventricular (icv) Cannula Implantation*

For *icv *treatment, animal were anesthetized with sodium pentobarbital (55 mg/kg bw, *ip*) and a 23 gauge thin-walled stainless steel guide cannula was stereotaxically implanted into the lateral cerebral ventricle according to the technique described in previous research.^22^ The sterotaxic coordinate with respect to the bregma were -0.8 mm posterior, -1.3 mm midline to lateral and 3.5 mm ventral. The guide cannula was fixed by three stainless steel screws and the mentioned screws were sealed with acrylic dental cement (pars acryl) in the target area. A dummy cannula with 30 gauges trimmed to the exact length of guide cannula and was inserted into the guide cannula. After surgical insertion of guide cannula and before the beginning of the experimental protocol, rats were allowed to recover for 7 days. During the recovery period, rats were brought to the experimental room and gently chandelled and removed their dummy cannula for 2 minutes. This process was done three times a day.^22^

*Histological Confirmation of Cannula Placement*

For the verification of guide cannula placement into lateral ventricles, two methods were used, (i) the presence of cerebrospinal fluid in guide cannula was confirmed and (ii) at the completion of each experiment, intracerebroventricular injection of methylene blue) 5 μl/rat) was followed by anesthetization of rats by pentobarbital. Rats were then euthanized by decapitation. Brain tissue was removed and visualization of methylene blue in the lateral ventricles confirmed that the guide cannula was correctly inserted. Data for four rats, where the guide cannula had not been inserted correctly, was omitted from our study analysis. 

*Induction of Morphine Dependency*

Morphine dependency was induced in 8 separate groups by subcutaneous injection of morphine in a dose-increasing manner (15-55 mg/kg) for 5 consecutive days.^22^

*Induction and Evaluation of Morphine Withdrawal Syndrome*

In order to induce withdrawal symptoms, all rats in each group were injected by naloxone 3 mg/kg and behaviors of each animal were recorded by a camera for a period of 60 minutes. The recorded behavior included jumping, head shake, wet dog shake, forepaw tremor, writhing, walking sniffing, sniffing, penile liking, rearing, chewing, body grooming, face wiping, swallowing, and teeth chattering. The number of each behavior was divided by their weighing factor (table 1) and a value was obtained. The summation of these values gave the total withdrawal score (TWS). These behavioral syndromes were commutated according to the previous studies.^22^

**Table 1:** Weighing factors (WFs) of different withdrawal signs of morphine in the mouse

**Behavior**	**WF**	**Behavior**	**WF**
Jumping	4	Body grooming	10
Head shake	5	Face wiping	10
Wet dog shake	5	Swallowing	10
Paw tremor	5	Teeth chattering	10
Writhing	5	Dysphoria	10
Walking sniffing	5	Rearing	20
Sniffing	5	Chewing	20
Penile liking	5	--------	--------

*Measuring the Blood Cortisol*

On the 10^th^ day, after all behavioral signs had been recorded; whole blood was collected from all animals. Serum was separated from whole blood and serum cortisol levels were measured in μg/dl by the ELISA method.

*Data Analysis*

All data obtained by this study were statistically analyzed using GraphPad Prism V6 software. The data was averaged in every experimental group and expressed as means±standard error of the means (SEM). In the next step, differences between controls and treatment groups were evaluated by one way ANOVA. Differences between severities of behaviors in the treatment of each group were evaluated by Dunnett post hoc test. A value with P<0.05 was taken as statistically significant. 

## Results

*Total Withdrawal Score (TWS) in Controls Groups and Groups under Treatment by Systemic Administration of Ramelteon*

**Figure 1:**
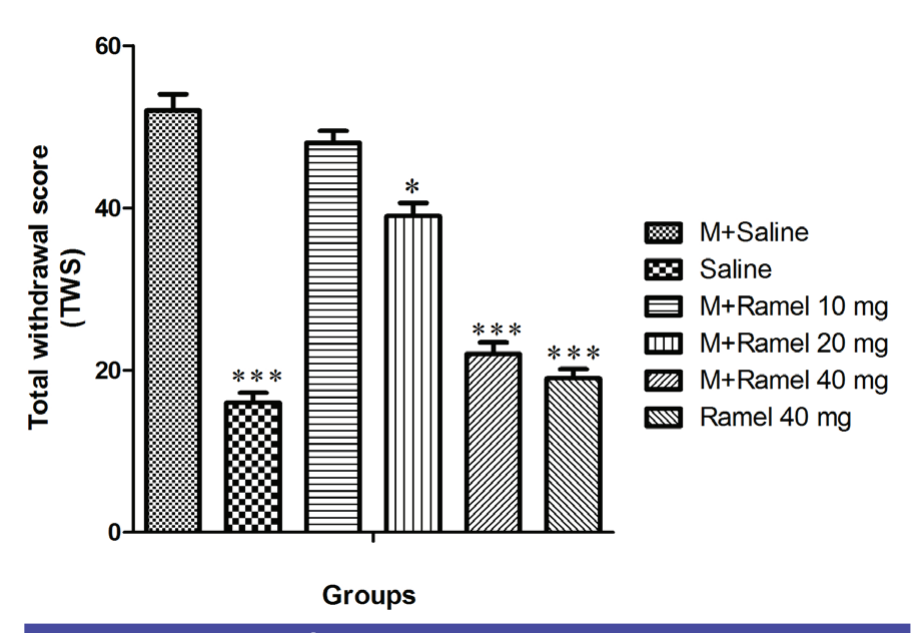
This figure shows total withdrawal score (TWS) in dependent group under treatment by systemic administration of ramelteon (10, 20, 40 mg/kg, bw), negative control group and ramelteon treatment group (40 mg/kg, bw) compared with positive control group. Data are expressed as the mean±SEM; ***P<0.001 and *P<0.05 different from positive control group; M: Morphine, Ramel: Ramelteon

The result of our study indicates that TWS in the negative control group (group I) during the experimental period was 16±1.2 while for positive control group (group II) it was 52±2 (P<0.001) (figure 1).

**Figure 2:**
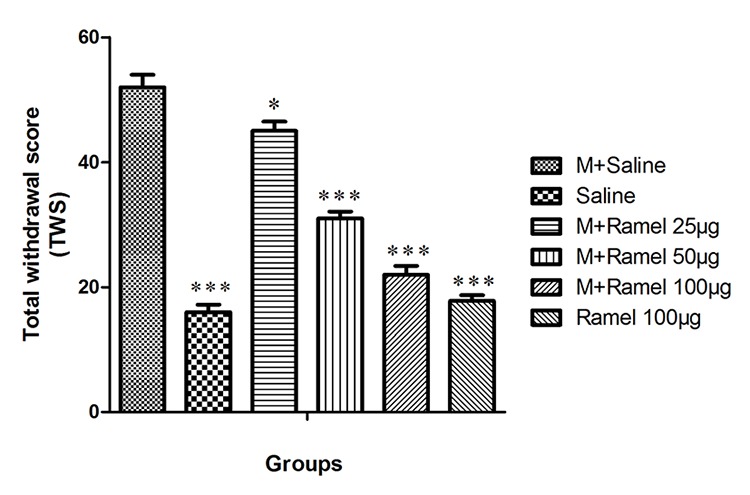
This figure shows total withdrawal score (TWS) in dependent group under treatment by central administration of ramelteon (25, 50 or 100 μg/5 μl/rat, icv), negative control group and ramelteon treatment group (100 μg/5 μl/rat, icv) compared with positive control group. Data are expressed as the mean±SEM; ***P<0.001 and *P<0.05 different from positive control group; M: Morphine, Ramel: Ramelteon

Intraperitoneal administration of ramelteon in dosage of 10, 20, or 40 mg/kg (groups III, IV and V) caused TWS decreases of 48±1.5, 39±1.6 and 22±1.4, respectively. Decreases in TWS were statistically significant for all three groups (II, IV and V) with statistical significance of P<0.001, P<0.05 and P<0.001, respectively, as compared with the positive control group. Additionally, group X, which exclusively received 40 mg/kg of ramelteon intraperitoneally, showed significant decreases in TWS in comparison with the positive control group (figure 2).

*Total Withdrawal Score (TWS) in Controls Groups and Groups under Treatment by Central Administration of Ramelteon*

TWS in the negative control group in which received normal saline during the experimental period was 16±1.2 while for the positive control group (group VI) TWS rate was 53±1.5 (P<0.001) (figure 2).

Intracerebroventricular injection of ramelteon in a dosage of 25, 50, or 100 μg/5 μl/rat (groups VII, VIII and IX) decreased the TWS to 45±1.5, 31±1.1 and 22±1.4, respectively. This attenuation in the group under treatment at the dose of 25 μg/5 μl/rat was statistically significant with P<0.05, and in groups under the treatment with doses of 50 or 100 μg/5 μl/rat were statistically significant with P<0.001 in comparison with the positive control group (group VI). Also, group XI, which received intracerebroventricular ramelteon at a dose of 100 ug/5ul/rat, showed significant decreases in TWS (17.8±0.9) in comparison with group VI, the positive control group (P<0.001) (figure 2).

*Effect of Systemic Administration of Ramelteon on Blood Cortisol Levels*

**Figure 3:**
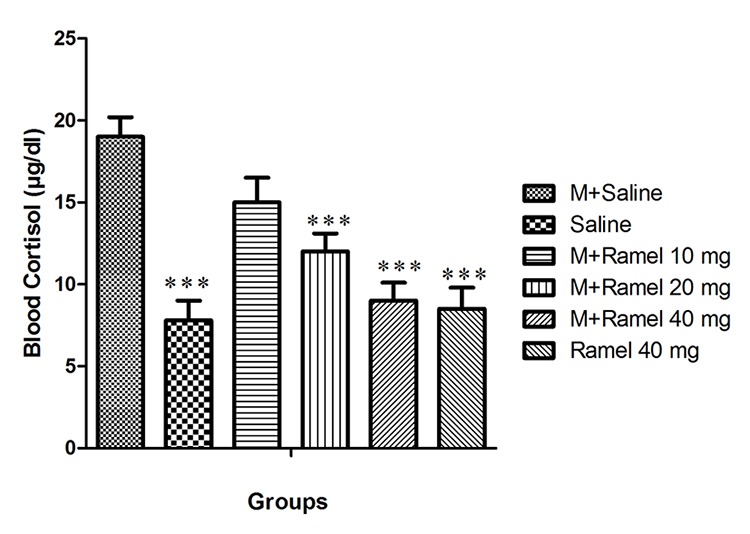
This figure shows blood cortisol level after the injection of naloxone in dependent group under treatment by systemic administration of ramelteon (10, 20, 40 mg/kg, bw) and negative control group and ramelteon treatment group (40 mg/kg, bw) compared with positive control group. Data are expressed as the mean±SEM; ***P<0.001 different from control positive group; M: Morphine, Ramel: Ramelteon

Our study indicates that blood cortisol levels in the negative control group (group I) were 7.8±1.2 μg/dl, while in the positive control group (group II) it was 19±1.2 μg/dl (P<0.001) (figure 3).

Intraperitoneal injection of ramelteon with doses of 10, 20, or 40 mg/kg (groups III, IV and V) caused attenuation of blood cortisol levels that reached 15±1.5, 12±1.1 and 9±1.1, respectively. Augmentation of blood cortisol levels were statistically significant (P<0.001) only in groups treated with doses of 20 or 40 mg/kg ramelteon. In the group under treatment with a ramelteon dose of 40 mg/kg (group X), blood cortisol levels were 8.5±1.3 and it was statistically significant in comparison with the positive control group (group II) (P<0.001) (figure 3).

*Effect of Central Administration of Ramelteon on Blood Cortisol Levels*

**Figure 4:**
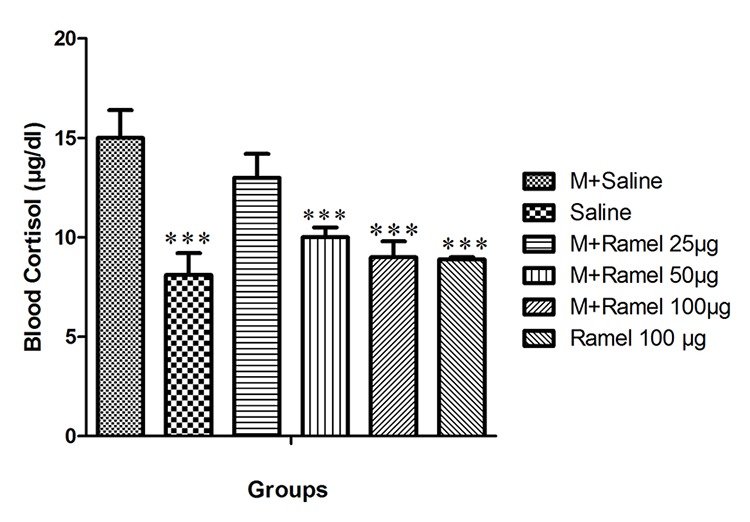
This figure shows blood cortisol level after the injection of naloxone in dependent group under treatment treated by central administration of ramelteon (25, 50 or 100 μg/5 μl/rat, icv) and negative control group and ramelteon treatment group (100 μg/5 μl/rat, icv) compared with positive control group. Data are expressed as the mean±SEM; ***P<0.001 different from control positive group; M: Morphine, Ramel: Ramelteon.

The blood cortisol level in the negative control group was 7.8±1.2 and was 15±1.4 for the positive control group (P<0.001) (figure 4).

Intracerebroventricular administration of ramelteon in doses of 25, 50 or 100 μg/5 μl/rat (groups VII, VIII and IX) attenuated the blood cortisol level to 13±1.2, 10±0.5 and 9±0.8, respectively. This attenuation in groups under treatment with doses of 50 or 100 μg/5 μl/rat was statistically significant with P<0.001 in comparison with the positive control group (group VI). Group XI, which received intracerebroventricular ramelteon at a dose of 100 ug/5 ul/rat, showed a significant decrease in blood cortisol levels (8.9±0.1) as compared with the positive control group (group VI) (P<0.001) (figure 4).

## Discussion

Characterized by withdrawal syndrome, long-term use of opioid derivatives, such as morphine, results in major health problems like dependency. As the results of our study indicate, the central administration of ramelteon in multiple dosages decreased the withdrawal symptoms significantly. Our experiments show that only high doses of systemic administration of ramelteon can attenuate withdrawal symptoms. Generally, our finding confirmed the idea that ramelteon central injection would attenuates morphine dependency. Previous study demonstrated that ramelteon, a non-selective melatonin receptor agonist, has a neuroprotective effects, and is used for the treatment of some neuroprotective effects and for the treatment of some neurodegenerative diseases such as Parkinson and Alzheimer.^8^^,^^23^^-^^25^ Previous study demonstrated that melatonin receptors have an antinociceptive activity in pain response; these results showed that melatonin has antinociceptive action by releasing of opioids like peptide in the brain region such as nucleus accumbens. Thus, because of this antinociceptive effect, this hormone could be beneficial in the attenuation of pain due to morphine withdrawal syndrome. The results of the present study indicate that, the total withdrawal score in central administration of ramelteon with multiple doses has a significant difference compared to the saline treatment group. However, the group that received only a high dose of systemic administration alone showed this type of attenuation. Thus, our study demonstrated that central administration is more effective in decreasing the withdrawal signs. Our study confirms the results of previous studies; in which pretreatment with melatonin significantly reduces the anxiolytic effects of rats experiencing withdrawal from repeated cocaine administration and that it can be used as a novel treatment for cocaine dependency.^14^^,^^19^^,^^26^^,^^27^ The physical withdrawal syndromes in morphine and cocaine dependent mice are inhibited by melatonin.^14^^,^^27^ Since ramelteon is metabolized by the cytochrome P-450 system, differences are seemed in the administration by the central method versus the systemic method. Due to the low metabolism of ramelteon, when it is administered centrally, the central administration can cause a greater reduction in morphine dependency as compared to the systemic administration. In addition, it has been indicated that ramelteon can protect dopaminergic cell from cytotoxicity of 6-hydroxy dopamine (6-HO). Furthermore, these studies have also demonstrated that ramelteon has an antioxidative properties.^28^^,^^29^

Previous studies have indicated that dopaminergic and serotonergic systems of the nucleus accumbens have a critical role in opioid dependence.^30-33^ These studies described interactions between pineal melatonin and both the serotonergic and dopaminergic systems, and suggested that melatonin has potential modulator effects on both of these systems; cell bodies and associated behavioral effects.^30^^,^^34^ In addition, the results of some other studies suggest that melatonin modulates the release of dopamine in the nucleus accumbens and plays a role in the reward system function in rats.^35^ This study demonstrated that ramelteon can protect dopaminergic, serotonergic and cholinergic neurons, all of which are involved in morphine dependency.^35^

Furthermore, morphine withdrawal syndrome is characterized by an increase in the hypothalamus–pituitary–adrenocortical (HPA) axis activity. Morphine dependence increased HPA axis activity with changes in corticotrophin releasing hormone (CRH) mRNA gene expression in selective neurons of the paraventricular nucleus.^36^ Our study demonstrates that central and systemic administration of ramelteon can attenuate blood concentrations of cortisol in withdrawal syndrome periods. Ramelteon seems to have anxiolytic effects, which could be beneficial in morphine withdrawal attenuation signs. Melatonin reduced postoperative anxiety and morphine consumption. Thus, melatonin receptor agonist, such as ramelteon, can reduce withdrawal syndrome anxiety and may be beneficial for morphine abandonment treatment. Other studies demonstrated anxiolytic properties of melatonin type-2 receptors.^37^ Our study showed melatonin has high potential for use as an anxiolytic agent for the management and stress-modulation of withdrawal syndrome.

## Conclusion

The results of this study show that systemic administration of ramelteon at a dose of 20 and 40 mg/kg significantly attenuates morphine dependence. Our study also shows that central administration of ramelteon at 25, 50 and 100 μg/5 ul can mitigate the total morphine withdrawal syndrome. Lastly, systemic administration of ramelteon at 20 and 40 mg/kg and central administration of ramelteon at 50 and 100 μg significantly attenuates blood cortisol levels during withdrawal episodes. All of these pharmacological actions may be relevant to the potential therapeutic effect of ramelteon on morphine physical dependence. However, further molecular investigations are required to clarify our results for the possibility of ramelteon in attenuating morphine physical dependency. 
